# Personalizing Elective Nodal Irradiation in Head and Neck Squamous Cell Carcinoma: A Systematic Review of SPECT/CT-Guided Sentinel Lymph Node Mapping

**DOI:** 10.3390/curroncol32120678

**Published:** 2025-12-01

**Authors:** Areti Gkantaifi, Nikolaos S Georgopoulos, Maria Rafaela Tezapsidou, Isidoros Ntioudis, Georgios Giakoumettis, Evanthia Giannoula, Emmanouil Papanastasiou, Argyrios Doumas, Ioannis Iakovou, Ioannis Aletras, Georgia Lymperopoulou, Maria Tolia

**Affiliations:** 1 Department of Radiation Oncology, School of Medicine, Faculty of Health Sciences, AHEPA University Hospital, Aristotle University of Thessaloniki, 54636 Thessaloniki, Greece; agkanta@auth.gr (A.G.); mariellatezapsidou@yahoo.gr (M.R.T.); 2 Fourth Department of Surgery, Attikon University Hospital, National and Kapodistrian University of Athens, 12462 Athens, Greece; 3 2nd Oncology Clinic of Theageneio Hospital, ‘Theageneio’ Cancer Hospital, 54639 Thessaloniki, Greece; isidorosntd@gmail.com; 4 Medical Physics and Digital Innovation Laboratory, AHEPA University Hospital, Aristotle University of Thessaloniki, 54636 Thessaloniki, Greece; ggiakoumettis@auth.gr (G.G.); empapana@auth.gr (E.P.); 5 First Academic Nuclear Medicine Clinic, School of Medicine, Faculty of Health Sciences, AHEPA University Hospital, Aristotle University of Thessaloniki, 54636 Thessaloniki, Greece; 6 Department of Radiotherapy, School of Medicine, University of Crete, 71500 Heraklion, Greece; aletras.ioannis@gmail.com; 7 Department of Radiology, Areteion Hospital, School of Medicine, National and Kapodistrian University of Athens, 76 Vasilissis Sofias Avenue, Ilisia, 115 28 Athens, Greece

**Keywords:** head and neck squamous cell carcinoma (HNSCC), radiotherapy, elective nodal irradiation (ENI), sentinel lymph node (SLN) mapping, single-photon emission computed tomography/computed tomography (SPECT/CT), lymphatic drainage mapping, quality of life, toxicity

## Abstract

Bilateral elective nodal irradiation (ENI) remains standard for treating most head and neck squamous cell carcinomas (HNSCC) but is associated with significant toxicity. Advances in lymphatic mapping, particularly with SPECT/CT-guided sentinel lymph node (SLN) identification, have enabled more personalized radiotherapy strategies. This systematic review evaluates the efficacy and quality-of-life impact of ENI strategies using SPECT/CT-guided SLN mapping. This systematic review, conducted according to PRISMA guidelines, included ten studies published between January 2014 and March 2024, including prospective, retrospective studies, randomized trials, and systematic reviews, examining oncologic outcomes and toxicity in patients undergoing SPECT/CT-guided SLN mapping or individualized ENI. Findings show that in well-lateralized, early stage carcinomas, SPECT/CT-guided ENI safely allows for unilateral treatment in up to 82% of patients, with a low contralateral regional failure rate. This approach significantly reduces radiation exposure to organs at risk and rates of xerostomia, dysphagia, and hypothyroidism, leading to improved quality of life. However, its applicability to advanced or midline tumors remains limited. SPECT/CT-guided SLN mapping and individualized ENI offer a promising, less toxic alternative for selected patients. Further prospective, multicenter, and randomized studies are needed to confirm these benefits and support broader clinical adoption.

## Simple Summary

Head and neck squamous cell carcinoma has high rates of lymph node involvement, significantly impacting prognosis. Sentinel lymph node biopsy, especially when combined with SPECT/CT imaging, offers a less invasive and accurate method for detecting occult metastases in clinically N0 patients. This approach enables more personalized elective nodal irradiation, allowing safe radiation de-escalation in well-lateralized, early stage carcinomas and potentially reducing radiation exposure and related toxicity, particularly xerostomia, dysphagia, and hypothyroidism, without compromising tumor control. However, further multicenter, randomized studies are needed to validate and standardize this approach in early stage carcinomas as well as in advanced or midline tumors.

## 1. Introduction

The GLOBOCAN database indicates that head and neck squamous cell carcinoma (HNSCC) ranks as the seventh most prevalent cancer globally, representing roughly 4.5% of all cancer diagnoses (890,000 new cases) and 4.6% of worldwide cancer fatalities (450,000 deaths) in 2020 [1]. The five-year survival rate for localized disease exceeds 86%, whereas locally advanced and metastatic disease are associated with significantly lower survival rates of approximately 69% and 40.4%, respectively.

Over 50% of patients with HNSCC exhibit regional lymph node involvement at diagnosis, attributable to the vast lymphatic network in the head and neck area [2]. Lymph node metastasis is strongly associated with poorer survival and remains a critical factor in treatment planning, as lymph node status serves as an independent prognostic indicator [3].

Retrospective studies have reported an overall incidence of occult lymph node metastasis in 20–30% of HNSCC cases, with variation by primary tumor site. Among patients with early stage (T1–T2) oral squamous cell carcinoma and clinically negative necks (cN0), the incidence ranges from 8.5% to 45%, averaging around 25.4% [4]. For laryngeal cancer, the incidence of nodal metastasis is estimated at 19.9% (95% CI: 16.4–23.4) for supraglottic tumors and 8.0% (95% CI: 2.7–13.3) for glottic tumors [5].

It is important to recognize that the indication and extent of elective nodal irradiation vary according to tumor subsite and biology. For instance, oral cavity squamous cell carcinoma (OSCC) typically exhibits predictable lymphatic drainage patterns, making unilateral ENI feasible in well-lateralized lesions. In contrast, HPV-positive oropharyngeal carcinoma (OPC) represents a distinct clinical and molecular entity characterized by higher nodal sensitivity but excellent prognosis, for which bilateral ENI remains standard in most protocols. Recognizing these variations is essential when interpreting SPECT/CT-guided mapping studies. Therefore, the concept of individualized ENI must be interpreted within the specific anatomical and biological context of each primary site. Multiple guideline groups, including DAHANCA, EORTC, ESTRO, and NCCN, have long supported bilateral ENI as standard for most head and neck subsites. However, accumulating radiotherapy evidence—such as the reports by Al-Mamgani et al. (*Eur J Cancer* 2017) [6] and Nuyts et al. (*Head Neck* 2021) [7]—demonstrates that unilateral ENI can be oncologically safe in well-lateralized oral cavity, tonsillar, and lateral wall hypopharyngeal cancers. These findings have prompted increasing interest in advanced imaging techniques, including PET/CT and SPECT/CT, to optimize nodal target definition and minimize normal tissue toxicity.

Several anatomical sub-sites in the head and neck, such as the soft palate, base of tongue, oral tongue, larynx, hypopharynx, and nasopharynx, exhibit crossing lymphatic drainage [8,9]. Since nodal involvement is a major prognostic factor, bilateral neck irradiation is typically recommended in international guidelines [10]. However, these recommendations do not often account for advances in modern imaging, which have substantially improved the accuracy of nodal staging and reduced the burden of occult disease in clinically negative lymph nodes [11].

Sentinel node biopsy (SNB) has emerged in HNSCC as a less invasive alternative to elective neck dissection, offering lower morbidity [12]. SNB facilitates the detection of occult metastases in head and neck cancer and provides a potential strategy for managing the clinically N0 neck. Incorporating SNB results into treatment planning enables more individualized prophylactic nodal irradiation volumes [13]. Personalized neck irradiation, guided by SNB findings, may allow for the omission of elective nodal irradiation (ENI) on one or both sides of the neck in up to 90% of patients, thereby reducing radiation-induced toxicity and improving quality of life without compromising oncologic safety [9]. The SNB procedure typically involves dynamic and static imaging after the administration of methylene blue and/or technetium-99m, followed by surgical removal of the identified sentinel nodes. Sentinel nodes can be localized by imaging within one hour of injection and are marked for intraoperative identification and excision [14].

The largest cohort study in oral cancer, the SENT trial, demonstrated that SNB detected occult metastasis in 23% of cases [13]. Notably, 85% of patients with positive sentinel nodes had no additional metastases found during neck dissection, and in 12.4% of cases, sentinel nodes were identified on the contralateral neck. SNB has been validated for staging T1–T2 cN0 oral cancers and is increasingly considered as an alternative to elective neck dissection [12]. A new meta-analysis on earlystage oral squamous cell carcinoma indicated a pooled sensitivity of 87% and a negative predictive value of 94% for sentinel node biopsy in identifying concealed nodal illness. In the case of most oral malignancies, sentinel lymph node biopsy (SNB) is regarded as a secure and precise alternative to elective neck dissection, with the exception of tumors located on the floor of the mouth [15].

While bilateral neck irradiation remains standard for HNSCC, studies indicate that the incidence of contralateral regional failure (CRF) in patients receiving unilateral irradiation is less than 10% [16]. Unilateral ENI appears to be justified in well-lateralized tumors without extension beyond the midline, as midline involvement is a key risk factor for CRF [17].

Single-photon emission computed tomography/computed tomography (SPECT/CT) is an appealing technique that may help individualize ENI volumes in carefully selected patients, thereby reducing radiation exposure to critical organs and minimizing treatment-related morbidity, including xerostomia, dysphagia, and hypothyroidism [18].

The aim of this literature review is to evaluate the safety and utility of sentinel lymph node (SLN) mapping with SPECT/CT in patients with clinically N0 HNSCC and to inform individualized elective nodal irradiation approaches. This review will highlight the impact of these strategies on tumor control and radiation-related toxicity, with the goal of optimizing both oncologic and quality-of-life outcomes.

## 2. Materials and Methods

**Figure 1:**
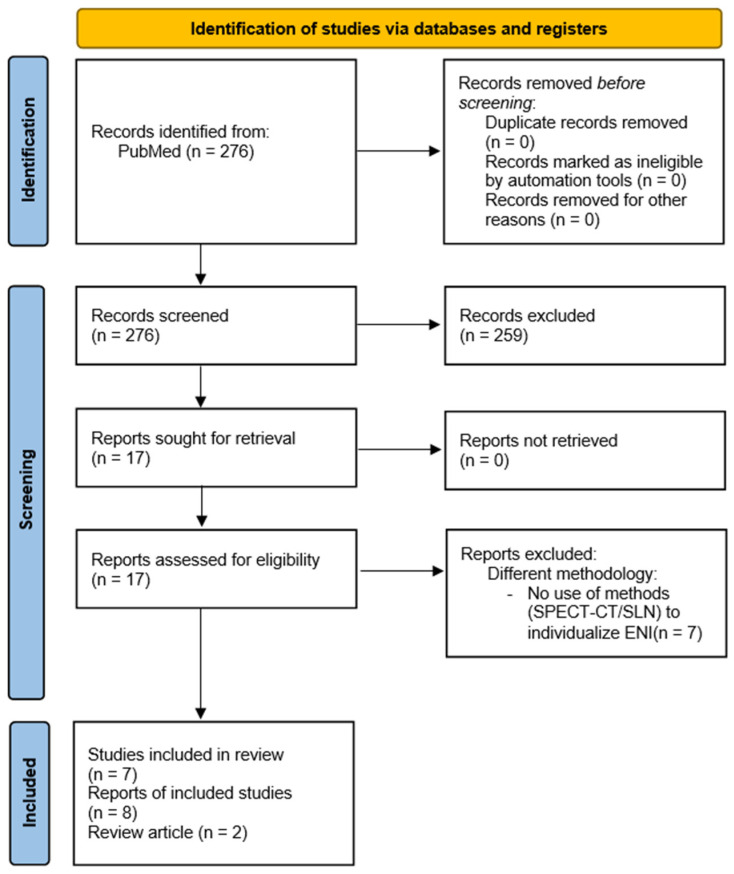
PRISMA 2020 flow diagram for new systematic reviews which included searches of databases and registers only. *From:* Page, M.J.; McKenzie, J.E.; Bossuyt, P.M.; Boutron, I.; Hoffmann, T.C.; Mulrow, C.D.; Shamseer, L.; Tetzlaff, J.M.; Akl, E.A.; Brennan, S.E.; et al. The PRISMA 2020 statement: An updated guideline for reporting systematic reviews. *BMJ* **2021**, *372*, 71. https://doi.org/10.1136/bmj.n71 [19]. For more information, visit: http://www.prisma-statement.org/.

This systematic review was conducted in compliance with the Preferred Reporting Items for Systematic Reviews and Meta-Analyses (PRISMA) guidelines (See also Figure 1) [19]. A thorough search of the PubMed database was conducted to locate publications published from January 2014 to March 2024 that assessed the application of SPECT/CT-guided sentinel lymph node mapping and personalized elective nodal irradiation in head and neck squamous cell cancer (HNSCC). The search technique integrated the subsequent keywords and MeSH terms: “head and neck cancer,” “sentinel node,” “lymph drainage mapping,” “SPECT/CT,” “unilateral elective irradiation,” and “bilateral elective irradiation.”

Eligible studies met the following criteria: (1) prospective or retrospective clinical studies, randomized controlled trials, or systematic reviews; (2) included adult patients (≥18 years) with histologically confirmed HNSCC; (3) evaluated SPECT/CT-guided sentinel node mapping, lymphatic drainage mapping, or personalized elective nodal irradiation; (4) reported oncologic outcomes (e.g., regional control; recurrence rates), radiation-induced toxicity, or quality of life; and (5) were published in English. The exclusion criteria encompassed the following: (1) case reports, editorials, letters, conference abstracts, or non-peer-reviewed publications; (2) studies solely focused on surgical management without addressing radiotherapy planning or outcomes; (3) studies that did not employ SPECT/CT or relevant lymphatic mapping techniques; and (4) pediatric populations. In this review, the term ‘lymphatic mapping’ refers to the imaging—based visualization of sentinel lymph nodes and drainage pathways using SPECT/CT, independent of whether surgical biopsy was subsequently performed. In some studies, mapping served solely for radiotherapy planning (functional imaging-guided ENI), while others integrated it with sentinel lymph node biopsy (SLNB).

All titles and abstracts identified in the initial search were screened independently by two reviewers. Full texts of potentially eligible studies were retrieved and assessed for inclusion. Discrepancies were addressed through consensus or by consulting a third reviewer.

A standardized data extraction form was utilized to gather information on study design, patient demographics, tumor characteristics, imaging and radiotherapy techniques, endpoints (oncologic outcomes, toxicity, and quality of life), as well as key findings. Data were summarized descriptively, with quantitative outcomes tabulated where appropriate. Due to heterogeneity in study designs and reported outcomes, a narrative synthesis was performed.

The risk of bias for included studies was assessed using the Newcastle–Ottawa Scale for cohort and case-control studies and the Cochrane Risk of Bias tool for randomized controlled trials. Systematic reviews were appraised using the AMSTAR 2 checklist. The quality and strength of evidence were considered in the interpretation of findings.

## 3. Results

A PubMed search identified 10 relevant articles for this review, including 8 original studies and 2 reviews. The summary of these studies, evaluating SPECT/CT-guided elective nodal irradiation and sentinel node mapping in head and neck cancer, is shown below in Table 1.

In 2014, Daisne et al. conducted a phase I feasibility study involving 10 cN0 head and neck cancer patients, pioneering the use of SPECT/CT lymphoscintigraphy to localize sentinel nodes and personalize nodal target volumes [20]. All nodal levels containing the four hottest sentinel lymph nodes (SLNs) on SPECT/CT were included in the treatment plan. On average, 2.9 SLNs were identified per patient. The absence of a thermoplastic mask for SPECT/CT led to localization difficulties in some cases. Notably, the clinical target volume (CTV) and planning target volume were reduced by half compared to standard international guidelines (*p* = 0.006), resulting in significant dose reductions to organs at risk. The authors concluded that the approach was feasible, warranting further validation in phase II studies.

Building on these findings, Longton et al. (2020) conducted a prospective phase I–II study enrolling 34 additional cN0 head and neck cancer patients, using SPECT/CT-guided ENI to spare normal tissues [21]. This trial administered prophylactic irradiation to neck levels encompassing up to the four most thermally active sentinel lymph nodes (SLNs). Unilateral lymphatic drainage was observed in 48% of patients, with only one case contralateral to the primary tumor. Bilateral drainage was present in 40% of oropharyngeal cancer patients. Among eight recurrences, only one (2.3%) occurred in a non-irradiated nodal area. Median doses to organs at risk, especially contralateral salivary glands and the thyroid, were significantly reduced. NTCP models demonstrated median reductions in the risk of xerostomia (0.3–13.7%), dysphagia (1.7–10.8%), and hypothyroidism (14.0–36.1%), alongside improved QoL at six months, particularly in patients who received unilateral irradiation.

De Veij Mestdagh et al. treated forty patients with primary HNSCC (T1–3N0–2bM0) based on lymph drainage mapping using SPECT/CT [22]. Thirty-two patients with only ipsilateral lymph drainage received unilateral ENI, while eight with limited contralateral drainage were treated to the corresponding contralateral neck level. Compared to standard bilateral irradiation, significant median dose reductions were achieved for the contralateral parotid gland (19.2 Gy), submandibular gland (27.3 Gy), laryngeal structures, constrictor muscles, and thyroid gland (all *p* < 0.001). Median reductions in NTCP were observed for xerostomia (19.7%), parotid function (13.5%), dysphagia (10.2%), hypothyroidism (20.3%), and laryngeal edema (4.5%). The largest benefits were observed in the subset treated with unilateral ENI.

From 2015 to 2017, the SUSPECT study (de Veij Mestdagh et al.) prospectively evaluated 50 patients with lateralized HNSCC (T1–3N0–2bM0) using SPECT/CT-guided ENI [23]. Of these, 82% exhibited ipsilateral-only lymphatic drainage and were managed with unilateral ENI. The remaining 18% had drainage to a single contralateral neck level and received targeted contralateral irradiation. Contralateral drainage was most commonly identified in level II. After a median follow-up of 33 months, the two-year incidence of contralateral regional failure (CRF)—the primary endpoint—was 2% (95% CI: 0–6%). Relative to a matched cohort receiving bilateral ENI, patients in the SPECT/CT-guided group exhibited higher quality of life and significantly lower rates of grade two dysphagia (54% vs. 82%, *p* < 0.001), tube feeding (10% vs. 50%, *p* < 0.001), and late grade two xerostomia (9% vs. 54%, *p* < 0.001).

The ongoing SUSPECT-2 trial is a one-armed, single-center prospective study including patients with primary T1–4N0–2b HNSCC not crossing the midline [24]. Unlike the original SUSPECT study, SUSPECT-2 incorporates contralateral sentinel node procedures for patients with contralateral lymphatic drainage identified on SPECT/CT. Patients without contralateral drainage or pathological sentinel nodes receive unilateral ENI. The primary outcome is the incidence of contralateral regional failure in the first two years, with secondary endpoints including radiation-induced toxicity and QoL. Results are pending.

Novikov et al. (2021) employed SPECT/CT to identify sentinel lymph nodes in 26 individuals diagnosed with oral tongue cancer [25]. Bilateral lymphatic drainage was detected in 38.5% of cases; unilateral drainage was seen in 61.5%, with no contralateral metastases observed. Their modeling demonstrated that SPECT/CT-guided radiotherapy for unilateral drainage could reduce irradiated normal tissue volume by 59–70% and significantly decrease mean dose to the spinal cord and contralateral parotid gland. The authors concluded that lymph flow-guided target volume definition may personalize radiotherapy and substantially reduce exposure of organs at risk.

In a retrospective analysis, Berania et al. (2022) used preoperative SPECT/CT lymphatic mapping to guide surgical and adjuvant management in 13 patients with lateralized oral or oropharyngeal cancer not crossing the midline [26]. All underwent SPECT/CT followed by transoral robotic surgery and ipsilateral neck dissection. Four patients required postoperative radiotherapy: three with unilateral drainage received unilateral irradiation, and one with bilateral drainage received bilateral irradiation. No failures in the contralateral neck were seen; however, one patient experienced a local recurrence.

A review by Al-Mamgani et al. reported a mean contralateral regional failure rate of 2.4% (95% CI: 1.6–3.5%) after unilateral lymph node dissection in 1116 head and neck cancer patients, increasing to 12.1% in the presence of medial lesions [28]. The authors advocate unilateral ENI for well-lateralized oral cavity, oropharyngeal, and lateral wall hypopharyngeal tumors not approaching the midline.

Nuyts et al. (2021) critically reviewed the indications and benefits of unilateral and SPECT/CT-guided ENI in definitive radiotherapy for head and neck cancer, highlighting the findings and limitations of the SUSPECT and Longton studies [7]. The review expressed concerns regarding the reliability of SLN procedures in patients with more advanced tumors (≥T3), nodal involvement, floor-of-mouth tumors, and certain oropharyngeal, laryngeal, and hypopharyngeal cancers and emphasized the complementary role of PET/CT imaging.

A recent retrospective institutional study by Razavian et al. (2024) evaluated unilateral neck approaches in 71 patients with early T-stage tonsil carcinoma managed with surgery, radiotherapy, or combined modalities, with treatment volumes limited to the primary tumor and ipsilateral neck [27]. No contralateral neck failures were reported after a median follow-up of 27 months. At 2 years, overall survival, progression-free survival, and locoregional control were 92%, 85%, and 88%, respectively. These findings suggest that, in appropriately selected patients, unilateral treatment provides excellent disease control with a very low risk of contralateral failure, supporting further study of unilateral strategies in this population.

## 4. Discussion

The management of the clinically negative (cN0) neck in head and neck squamous cell carcinoma (HNSCC) remains a subject of ongoing refinement as clinicians seek to balance oncologic safety with minimization of treatment-related toxicity. Traditionally, bilateral elective nodal irradiation (ENI) has been the standard of care for most HNSCC patients, based on early evidence demonstrating improved regional control and overall survival. However, this approach is well recognized to increase the risk of both acute and late radiation-induced toxicities, notably xerostomia, dysphagia, and hypothyroidism, which can have a profound impact on quality of life (QoL).

Although the data summarized herein support de-escalation strategies in well-lateralized oral and tonsillar primaries, these findings may not directly apply to HPV-positive oropharyngeal, midline, or advanced T-stage tumors, where bilateral ENI remains the standard of care. Recent advances in lymphatic mapping and imaging, particularly the use of single-photon emission computed tomography/computed tomography (SPECT/CT) for sentinel lymph node (SLN) identification, have enabled more individualized approaches to ENI [28]. The justification for SPECT/CT-guided ENI is that it can avoid radiation exposure to unaffected nodal areas and adjacent organs at risk (OARs), especially in patients with well-lateralized tumors and no indication of contralateral lymphatic drainage [15].

Multiple prospective studies and cohort analyses now support the feasibility, safety, and QoL benefits of SPECT/CT-guided ENI. Daisne et al. first demonstrated that SPECT/CT lymphoscintigraphy could successfully identify SLNs for target delineation, resulting in a significant reduction in clinical and planning target volumes and, consequently, lower doses to OARs [20]. Building on this, Longton et al. and de Veij Mestdagh et al. reported in larger prospective series that SPECT/CT-guided ENI enabled a substantial proportion of patients—up to 82%—to receive unilateral neck irradiation, with marked reductions in median doses to contralateral salivary glands, laryngeal structures, and thyroid [21,22]. Importantly, these dosimetric enhancements resulted in significant decreases in the risk of xerostomia, dysphagia, and hypothyroidism, as anticipated by normal tissue complication probability (NTCP) models and corroborated by better patient-reported quality of life outcomes at follow-up [21].

The safety of this approach is further supported by the low rates of regional recurrence observed in these studies. In the SUSPECT trial, the 2-year contralateral regional failure rate was only 2%, comparable to historical series using bilateral ENI [12]. Similarly, Novikov et al. and Berania et al. found no contralateral recurrences in patients selected for unilateral irradiation based on SPECT/CT mapping, supporting the notion that careful patient selection can preserve disease control while reducing treatment morbidity [25,26]. These findings align with the review by Al-Mamgani et al., which reported a mean contralateral regional failure rate of 2.4% for unilateral approaches in well-lateralized tumors, though this risk increased to over 12% for medial or midline lesions—highlighting the importance of appropriate case selection [6,28].

The findings from Razavian et al. further reinforce the safety of unilateral neck management in carefully selected patients with early-stage, well-lateralized tonsil carcinoma [29]. In their cohort, no contralateral neck failures were observed, and survival and locoregional control outcomes were excellent, regardless of whether patients received surgery, radiotherapy, or both. These results, consistent with prior SPECT/CT-guided and imaging-based studies, suggest that unilateral approaches can maintain oncologic safety while reducing treatment burden and toxicity [7]. This growing body of evidence strengthens the rationale for prospective trials and broader adoption of individualized elective nodal management in selected head and neck cancer patients.

Nonetheless, there are notable limitations and areas of ongoing debate. The reliability of SLN mapping and SPECT/CT guidance in tumors with more advanced stage (≥T3), nodal involvement, floor-of-mouth location, or poorly lateralized oropharyngeal, laryngeal, and hypopharyngeal tumors remains uncertain [30]. Nuyts and others have emphasized the need for caution and further validation in these subsets, as well as the potential complementary role of advanced imaging modalities, such as PET/CT, for more accurate staging [7].

These observations are consistent with recent modeling and clinical studies that emphasize patient-specific ENI based on tumor lateralization and lymphatic geometry. Ludwig et al. introduced a probabilistic model quantifying contralateral lymphatic spread risk, providing a framework for patient-specific ENI adaptation, and demonstrated that contralateral spread probability can be accurately predicted using individualized lymphatic-flow simulations [31]. Gau et al. demonstrated oncologic safety of unilateral irradiation in well-lateralized tumors and reported excellent regional control and significant toxicity reduction using imaging-guided unilateral ENI [32]. Similarly, a more recent review underscored the growing consensus toward ENI de-escalation supported by SPECT/CT and PET-based imaging [33]. Together, these data strengthen the evidence base supporting functional-imaging-guided and site-adapted ENI strategies now being tested in ongoing prospective trials.

Ongoing studies, including the SUSPECT-2 trial, are expected to provide further evidence regarding the long-term safety and clinical utility of this strategy, particularly in broader patient populations and with more robust endpoints for quality of life and late toxicity [24].

In summary, SPECT/CT-guided elective nodal irradiation and sentinel node mapping represent promising strategies for individualizing radiotherapy in HNSCC. In carefully selected patients—primarily those with well-lateralized, earlystage tumors and no evidence of contralateral lymphatic drainage—this approach can substantially reduce radiation exposure to healthy tissues, lower the risk of treatment-related morbidity, and improve quality of life, without compromising oncologic outcomes. These findings support the consideration of personalized elective nodal irradiation as a new standard in selected cases, while underscoring the need for continued research and multidisciplinary evaluation in broader clinical contexts.

Looking ahead, several important questions remain to be addressed as the field moves toward more personalized approaches in the management of the clinically negative neck in head and neck cancer. Large, multicenter, and randomized studies are needed to confirm the long-term oncologic safety and late toxicity profiles of SPECT/CT-guided and sentinel node-based elective nodal irradiation strategies. It remains to be determined whether individualized ENI can be safely extended to patients with more advanced tumors, such as those with ≥T3 oropharyngeal, hypopharyngeal, or laryngeal cancers, or with limited midline involvement. Further research is also warranted to define the optimal imaging modality—or combination of modalities—for reliably mapping lymphatic drainage and identifying sentinel nodes in various head and neck subsites.

Additionally, the impact of these individualized approaches on patient-reported outcomes and long-term quality of life should be explored in greater depth. The potential role of molecular or radiomic biomarkers in refining patient selection for unilateral versus bilateral ENI is another promising area for investigation. Evaluating the cost-effectiveness and resource implications of adopting advanced imaging and personalized irradiation protocols in clinical practice will be essential for broader implementation. Finally, fostering multidisciplinary collaboration and developing targeted educational and training initiatives will be critical in supporting the standardization and integration of SPECT/CT-guided elective nodal irradiation into routine care. Addressing these questions will help define the next steps for optimizing both oncologic outcomes and quality of life for patients with head and neck cancer.

## 5. Conclusions

Recent improvements in lymphatic mapping and imaging, including the utilization of SPECT/CT-guided sentinel node identification, have opened new avenues for customizing elective nodal irradiation in head and neck squamous cell carcinoma.

Evidence from feasibility studies, prospective trials, and cohort analyses consistently demonstrates that, in carefully selected patients with well-lateralized tumors and no contralateral lymphatic drainage, this approach can safely reduce the extent of irradiation, minimize toxicity, and improve quality of life without compromising regional control. While these findings are promising, further validation in larger, randomized studies is needed, particularly for patients with more advanced disease or challenging tumor locations. As the field progresses toward more tailored treatment strategies, multidisciplinary collaboration and ongoing research will be essential to fully realize the benefits of personalized radiotherapy for head and neck cancer.

SPECT/CT-guided SLN mapping and individualized or unilateral ENI are promising strategies for reducing treatment toxicity and improving quality of life in selected HNSCC patients, including those with tonsil carcinoma, without compromising regional control. Further prospective, multicenter, and randomized studies are required to validate these findings and standardize implementation in broader patient populations.

## Figures and Tables

**Table 1:** Summary of key studies evaluating SPECT/CT-guided elective nodal irradiation and sentinel node mapping in head and neck cancer, with a focus on oncologic safety, reduction in toxicity, and quality of life outcomes.

Study (Year)	Design	N (Patients)	TumorType/Stage	Technique	Main Findings/Outcomes
Daisne et al., 2014 [20]	Phase I-feasibility	10	cN0 HNSCC	SPECT/CT-SLN mapping for ENI	SPECT/CT-guided ENI halved CTV/PTV vs. standard; significant dose reduction to OARs; demonstrated feasibility
Longton et al., 2020 [21]	Prospective phase I–II	34	cN0 HNSCC	SPECT/CT-guided ENI	48% unilateral drainage; 2.3% relapse outside ENI; significant dose reduction to OARs; reduced NTCP for xerostomia, dysphagia, hypothyroidism
de Veij Mestdagh et al., 2018 [22]	Prospective cohort	40	T1–3N0–2bM0 HNSCC	SPECT/CT lymph drainage mapping	Unilateral ENI in 32/40; significant median dose/NTCP reduction for OARs; improved QoL, largest benefit in unilateral ENI group
SUSPECT, 2020 [23]	Prospective single-arm	50	Lateralized T1–3N0–2bM0 HNSCC	SPECT/CT-guided ENI	82% unilateral drainage (unilateral ENI); 2% (1/50) contralateral failure at 2 yrs; improved QoL and lower toxicity vs. bilateral ENI
SUSPECT-2 (Ongoing) [24]	Prospective single-arm	Planned > 50	T1–4N0–2b HNSCC, lateralized	SPECT/CT + contralateral SLN mapping	Endpoint: contralateral failure at 1–2 yrs, toxicity, QoL. Results pending
Novikov et al., 2021 [25]	Prospective cohort	26	Oral tongue cancer	SPECT/CT SLN mapping	38.5% bilateral, 61.5% unilateral drainage; no contralateral metastases; modeling shows >50% OAR dose/volume reduction with personalized ENI
Berania et al., 2022 [26]	Retrospective cohort	13	cT1–2N0 lateralized OPSCC	SPECT/CT lymphatic mapping	All had SPECT/CT mapping & surgery; 4 needed adjuvant RT; no contralateral failures; 1 local recurrence
Razavian et al., 2024 [27]	Retrospective cohort	71	Early T-stage tonsil carcinoma	Unilateral surgery and/or RT	2-yr OS 92%, PFS 85%, LRC 88%; no contralateral failures; supports safety of unilateral approaches
Al-Mamgani et al., 2017 [28]	Literature review	1116 (total patients reviewed across studies)	HNC, various types	Review of ENI (uni/bilateral)	Mean contralateral failure 2.4%; up to 12.1% for midline lesions; supports unilateral ENI for well-lateralized tumors
Nuyts et al., 2021 [7]	Narrative review	—	HNSCC	Literature review	Summarizes evidence for/against SPECT/CT-guided ENI; notes limitations in ≥T3, N+, FOM tumors; highlights role of PET/CT

Abbreviations: ENI = elective nodal irradiation; SLN = sentinel lymph node; SPECT/CT = single-photon emission computed tomography/computed tomography; HNSCC = head and neck squamous cell carcinoma; OARs = organs at risk; NTCP = normal tissue complication probability; QoL = quality of life; OPSCC = oropharyngeal squamous cell carcinoma; FOM = floor of mouth. Legend: ‘Mapping’ denotes SPECT/CT-based visualization of sentinel lymph nodes or drainage pathways, which may or may not include subsequent surgical biopsy depending on the study protocol.

## Data Availability

No new data were created or analyzed in this study.
